# Case Report: Robot-assisted laparoscopic nephron-sparing surgery for a renal abscess mimicking a tumor

**DOI:** 10.3389/fonc.2022.1027571

**Published:** 2022-10-31

**Authors:** Shun Wang, Xiangyi Liang, Di Pan, Jianqing Zhang, Kun Chen, Kehua Jiang, Tao Li

**Affiliations:** ^1^ Department of Urology, Guizhou Provincial People’s Hospital, Guiyang, China; ^2^ Zunyi Medical University, Zunyi, China; ^3^ Prenatal Diagnosis Center, Guizhou Provincial People’s Hospital, Guiyang, China; ^4^ Department of Urology, Affiliated Hospital of Guizhou Medical University, Guiyang, China

**Keywords:** renal abscess, renal tumor, antibiotic therapy, RA-NSS, distinguish

## Abstract

The differential diagnosis of renal tumors and abscesses is crucial owing to their different treatments. Although antibacterial administration and radiological examination are excellent means for distinction, misdiagnosis is common and may lead to severe consequences, such as the need for nephrectomy. Here, we report a case involving a 52-year-old Asian woman with a renal mass for which a differential diagnosis was challenging. The mass persisted after administration of intravenous antibiotic therapy for 1 month. A computed tomography scan indicated an inflammatory lesion, whereas magnetic resonance imaging suggested a diagnosis of a tumor. Despite these indications, a right renal abscess was suspected during robot-assisted laparoscopic surgery, and nephron-sparing surgery was performed, which allowed confirmation of the final pathological result by biopsy specimen. Postoperatively, the mass gradually decreased in size after antibiotic therapy for a further month. This case, in which a renal abscess mimicked a tumor and the patient almost underwent a nephrectomy, highlights the need for caution in establishing therapeutic schedules for patients with inaccurate diagnoses. The management strategies for such patients must be reviewed and improved.

## Introduction

Renal abscess is a subacute or chronic inflammatory disease that accounts for approximately 2% of all kidney diseases (1). Renal abscesses can be unilateral or bilateral and involve single or multiple lesions (2), and can be caused by hematogenous infection, pyelonephritis, and retrograde infection. They can manifest as gross hematuria, flank pain, fever, and altered blood examination results (elevated blood leukocytes, neutrophils, and erythrocyte sedimentation) (3, 4). Kidney tumors comprise 3% of all adult malignancies, with 210,000 new diagnoses each year in developed countries (5, 6). Renal abscesses can be effectively treated by antibiotics, puncture or drainage, and surgery, while resection is the standard management for kidney tumors (7, 8). Thus, the accurate differentiation of a renal abscess from a tumor is essential to avoid unnecessary surgery. Technological developments and increasing experience have improved the differential diagnosis between renal abscesses and tumors; however, misdiagnoses do still occur. Eltahawy (9) reported on 11 patients who presented with an initial diagnosis of perinephric suppuration or renal abscess, while their final confirmed diagnosis was renal carcinoma.

Here, we present a case of a renal abscess that was difficult to distinguish from a tumor on contrast-enhanced computed tomography (CT) and magnetic resonance imaging (MRI). The patient opted to undergo surgery owing to lesion persistence following the administration of intravenous antibiotic therapy for 1 month. Fortunately, a renal abscess was considered perioperatively and robot-assisted laparoscopic nephron-sparing surgery (RA-NSS) was performed.

## Case description

A 52-year-old Asian woman was admitted to a local hospital with a complaint of right flank pain that had appeared 1 month prior and had persisted for a few days. A renal mass was detected on contrast-enhanced CT, and the patient was prescribed an intravenous infusion of cefoxitin sodium (2 g, bid). The patient’s symptoms were completely alleviated when she was transferred to our department, but the renal lesion remained. Routine blood tests indicated a white blood cell count of 6.30 × 10^9^/L and a neutrophil percentage of 52.2%, while routine urine test results were as follows: white blood cells, 50/μl; red blood cells, 0/μl; C-reactive protein, 1.90 mg/L; and creatinine, 90 μmol/L.

**Figure 1:**
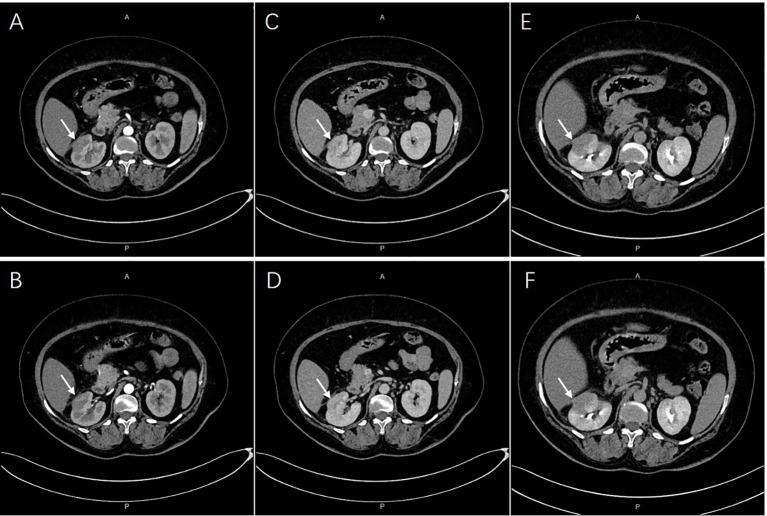
A multiple low-density lesions at right kidney. The enhancement degree was similar with renal parenchyma both in arterial [**(A, B)**, arrows] and venous phases [**(C, D)**, arrows]. Lower enhancement in excretory phase were imaged [**(E, F)**, arrows].

**Figure 2:**
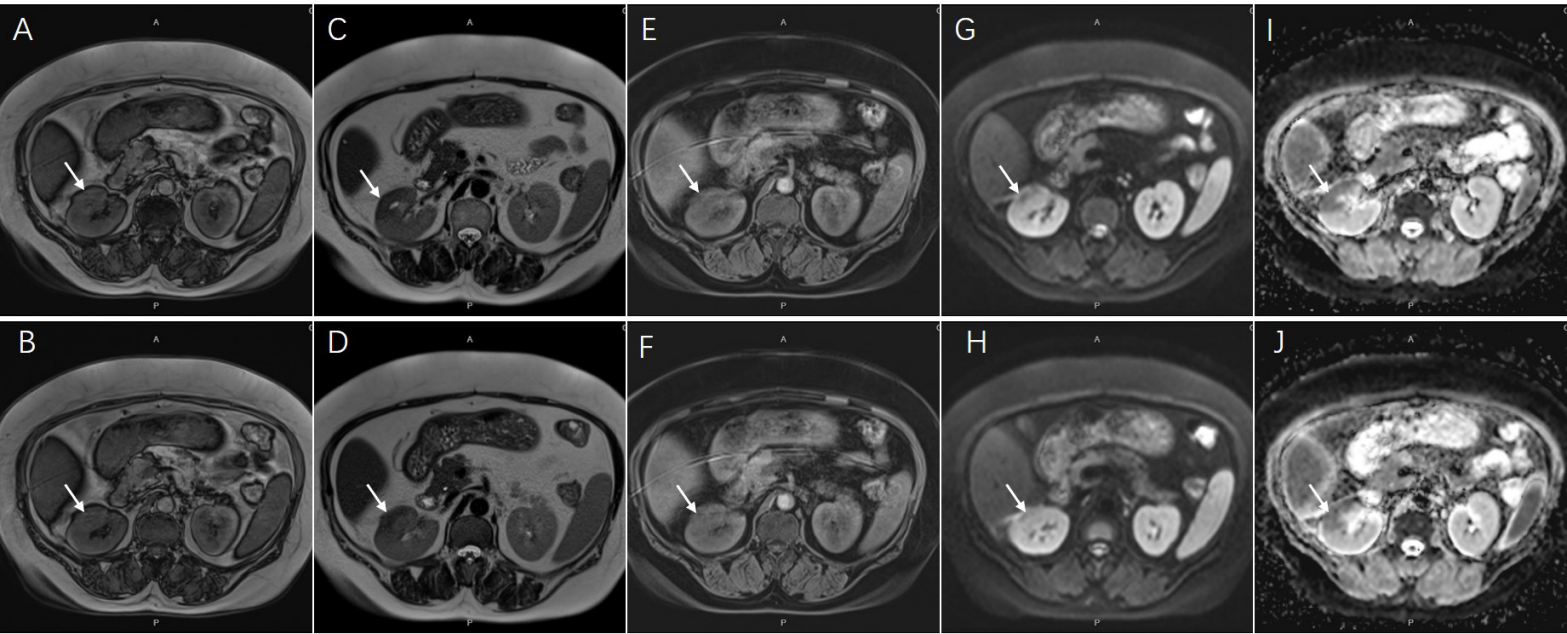
Magnetic resonance imaging showed an inhomogeneous lesion approximately 44x23mm in diameter [**(A, B)**, coronal T1-weighted image; **(C, D)**, coronal T2-weighted image, arrows]. The central area presented progressive enhancement in delayed phase [**(E, F)**, arrows]. The lesion also showed an opposite signal found in center on diffusion-weighted sequences [**(G, H)**, arrows]. The ADC map on corresponding areas presented low signal appearance [**(I, J)**, arrows].

Contrast-enhanced CT revealed a multiple low-density lesion with marginal haziness in the right kidney (**Figure 1**). The enhancement degree was similar to that of the renal parenchyma in both the arterial (**Figures 1A, B**) and venous (**Figures 1C, D**) phases but was lower in the excretory phase (**Figures 1E, F**). CT primarily indicated an inflammatory lesion, but a tumor was also considered. On MRI (**Figure 2**), T2-weighted imaging revealed a mass with low signal intensity and unclear margins in the exterior of the middle pole of the right kidney (**Figures 2C, D**). In the cortical phase, the enhancement degree was comparable between the lesion and the surrounding kidney cortex, while an area with a slightly lower signal was located in the center of the lesion. The central area showed progressive enhancement in the delayed phase, and the retroperitoneal lymph nodes were homogeneously enhanced (**Figures 2E, F**). A diffusion-weighted imaging sequence revealed patchy enhancement of the lesion, with a lower signal in the center (**Figures 2G, H**). In summary, MRI indicated a diagnosis of a renal tumor (oncocytoma or chromophobe cell carcinoma), although inflammatory lesions could not be excluded.

**Figure 3:**
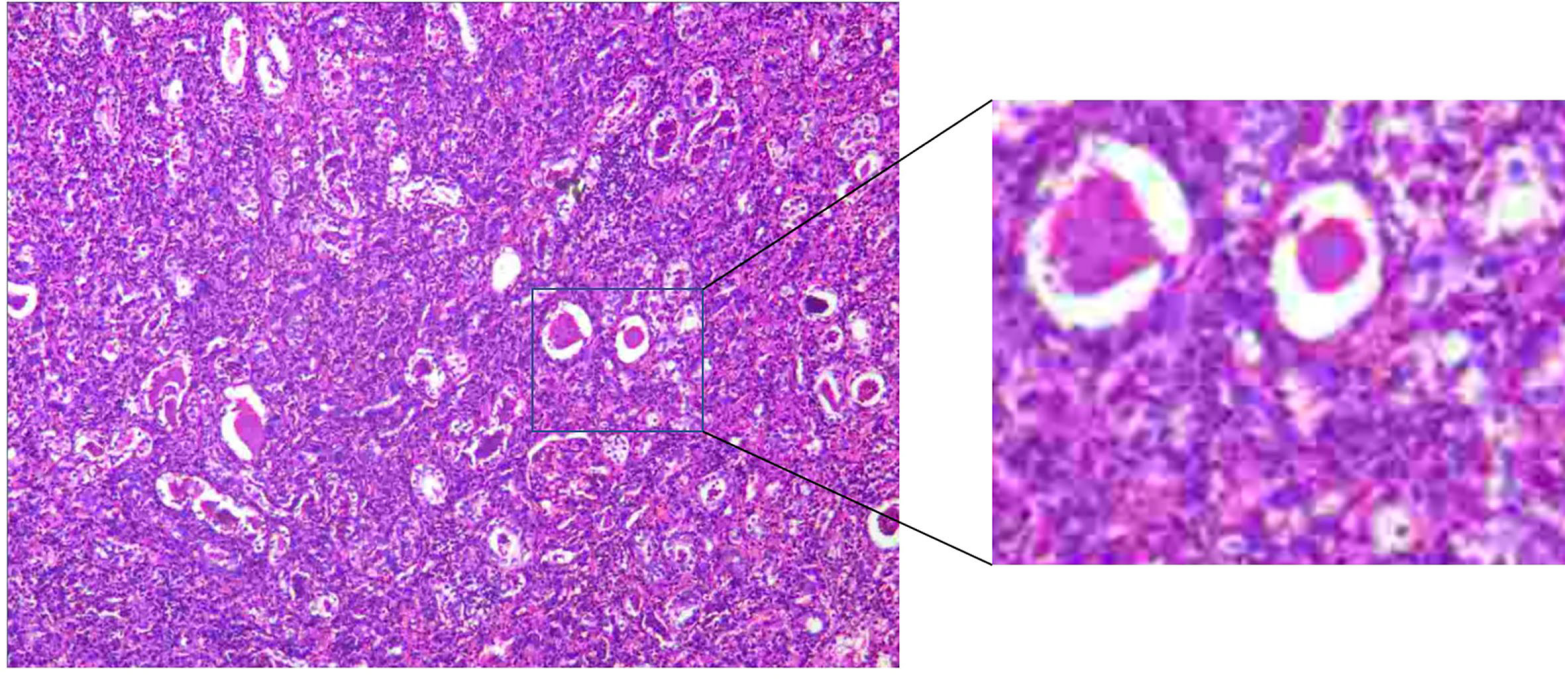
There were both acute and chronic inflammatory cells infiltration, compared with many small abscesses.

**Figure 4:**
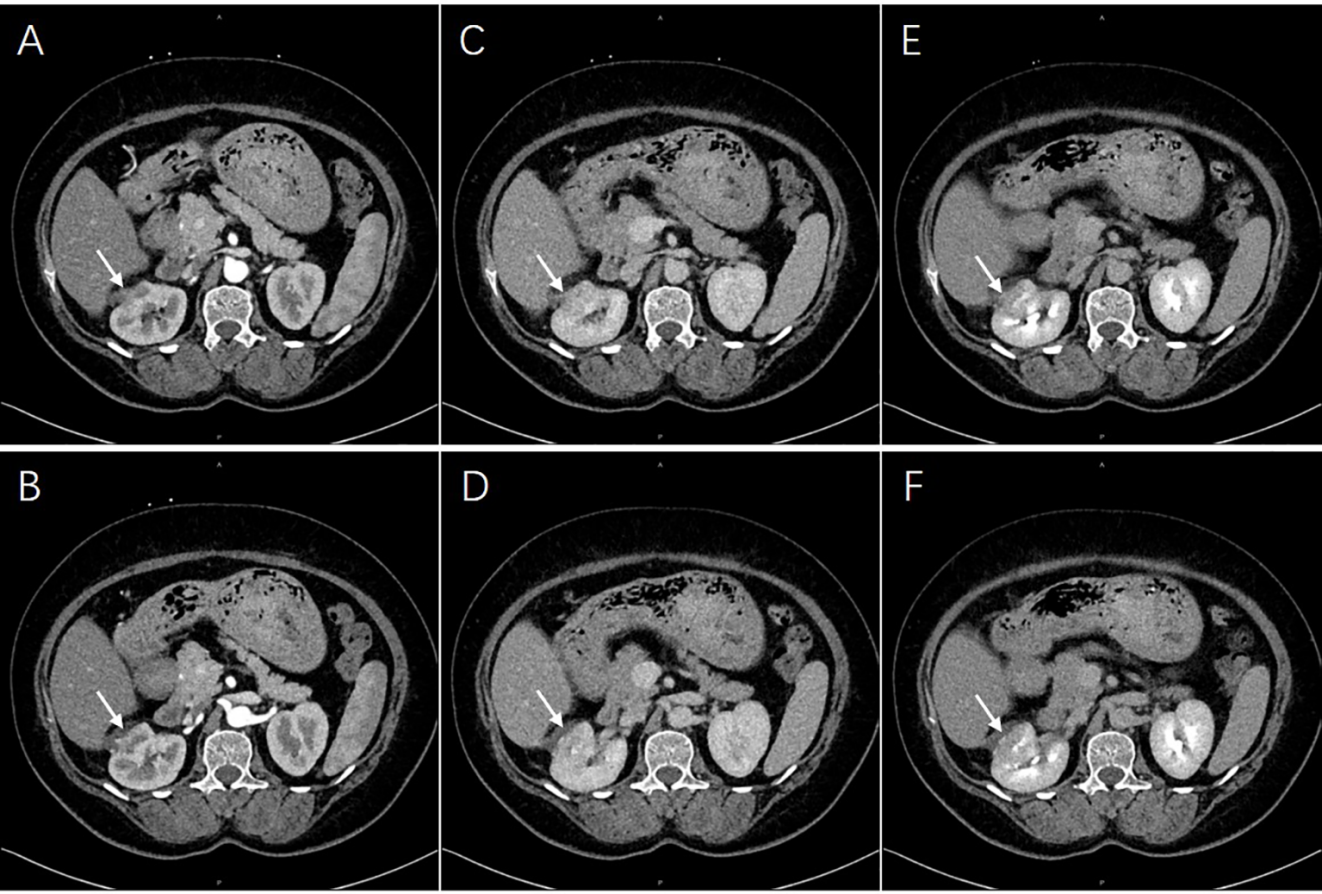
The lesions were diminished. They were enhanced equally with renal parenchyma in arterial [**(A, B)**, arrows] and venous phases [**(C, D)**, arrows], while less degree in excretory phase [**(E, F)**, arrows].

Based on the persistence of the lesion after administration of antibiotic therapy for 1 month, RA-NSS was planned because renal tumors could not be excluded. During the surgery, the upper pole of the right kidney was found to be surrounded by severe adhesions, accompanied by local edema and inflammatory exudation. After careful separation, no invasion of adjacent tissue was observed. Furthermore, the renal capsule was complete and there were no obvious lesions bulging on the renal surface. Consequently, the possibility of malignancy was low and tumor cells were not found by intraoperative freezing section. The final pathological result confirmed a renal abscess (**Figure 3**), and the lesion gradually diminished after one further month of antibiotic therapy with cefoxitin sodium (**Figure 4**).

## Discussion

Renal abscesses always occur in the perirenal cortex, where pathogenic bacteria can easily enter the subrenal capsule or penetrate the renal capsule into the perirenal space (10). The typical CT manifestation of renal abscesses is ring-shaped enhancement, but nodular, wall nodular, or irregular enhancement can also be found. Renal abscesses are also characterized by gas shadows in the abscess cavity, with a CT value between those of cysts and tumors (11, 12). The granulation tissue around an abscess may be enhanced on MRI, but the degree of enhancement is weaker, and the duration is longer compared with those of renal tumors (13, 14). For renal tumors, CT reveals isopycnic or mixed-density enhancement with some necrosis or calcification. They are also characterized by the enhancement of “fast-in and fast-out,” which is derived from the rich blood supply of the tumor (15). Using MRI, renal tumors exhibit equal or slightly lower signals in T1-weighted imaging and apparent diffusion coefficient (ADC) images, slightly higher and equal signals in T2-weighted imaging, and equal or slightly higher signals in diffusion-weighted imaging sequences compared with signals for surrounding non-cancerous tissues (16). CT and MRI are excellent at differentiating renal tumors from various diseases. However, these techniques indicated opposite diagnoses in the current case, which ultimately influenced the therapeutic strategy.

Alternative methods to CT and MRI are available, but there are limitations to these approaches and accurate identification of the nature of renal masses must remain the focus. Contrast-enhanced ultrasound presents an accuracy of 90%–96% in diagnosing renal malignancy (17); however, some benign kidney lesions are difficult to distinguish (18). Positron emission tomography (PET)/CT is also valuable, but some benign lesions present with higher metabolic levels than those of carcinomas (19, 20). In addition, Marshall (21) reported that the false-negative rate of PET/CT in diagnosing primary renal tumors is relatively higher and the sensitivity is not equal to that of CT. Renal biopsy is not regularly recommended for diagnosis because of the limitations of needle track implantation, puncture bleeding, and false-negative results (22–24). In summary, although there are numerous potentially effective methods available, the accurate distinction of renal tumors remains crucial, especially in cases like the present one.

Antibiotic therapy is the primary treatment for renal abscesses. Third-generation cephalosporins, penicillin, and aminoglycosides are effective against Gram-negative bacteria, and penicillinase-resistant penicillins are helpful against Gram-positive pathogens (25). The antibiotic must be adjusted based on urine or blood culture results and should be continued for several weeks until the infectious parameters return to normal (26). However, the precise duration for such antibiotic therapy has not been identified. In the present case, the patient’s symptoms were completely alleviated and the biochemical test results were almost normal after administering antibiotic therapy for 1 month; therefore, a urinary culture was not routinely performed.

For kidney abscesses, the lesion should reduce after long-term antibiotic therapy (27). However, the mass in the present case persisted despite 1 month of antibiotic management, and MRI primarily indicated a tumor. These observations challenged the recommendation of prolonged anti-infective treatment because this management strategy might have delayed tumor resection if renal malignancy was finally confirmed. Moreover, an operation strategy might have been overtreatment and could even have led to severe consequences if a renal abscess was definitively diagnosed. In the present case, the patient’s kidney was preserved *via* RA-NSS, but most renal abscesses or infectious lesions are consulted at junior hospitals where there is a lack of expertise, experience, and medical equipment. It is also a point of caution that a renal abscess can mimic a tumor even after long-term antibiotic therapy; thus, it is essential to accurately diagnose a renal mass.

The presence of a pseudocapsule has been reported in some cases of renal cell carcinoma, wherein the tumor was always accompanied by calcification, cystic degeneration, necrosis, and bleeding (28). Thus, renal tumors typically coexist with abscesses, which further highlights the need to monitor an abscess until it is completely resolved.

In the present patient, the imaging manifestation did not change after antibiotic therapy was administered for 1 month but diminished within 2 months of treatment (**Figure 4**). Thus, the optimal duration of antibiotic therapy should be considered for such patients to determine whether another course is necessary or suitable if a 1-month prescription is not effective. Moreover, although robotic surgery provides excellent therapeutic effects, future research should focus on strategies to obtain a definitive diagnosis and select appropriate treatment.

## Conclusion

The differential diagnosis between renal abscesses and tumors is challenging in some individuals. Antibiotic management for 1 month may be insufficient for selected patients, and prolonged antibiotic therapy should be considered, although the evidence on the appropriate duration of antibiotic therapy is insufficient. Performing surgery at the appropriate time is advisable, but an accurate diagnosis is more important for the selection of appropriate treatment and management.

## Data availability statement

The raw data supporting the conclusions of this article will be made available by the authors, without undue reservation.

## Ethics statement

Written informed consent was obtained from the participant for the publication of this case report.

## Author contributions

SW, XL and DP obtained and analyzed the clinical data. SW, JZ and TL wrote the manuscript. KC and XL designed and constructed the figures. KC, KJ and TL designed the study, contributed to study supervision, and edited the manuscript. All authors contributed to writing and revising the manuscript and figures. All authors contributed to the article and approved the submitted version.

## Funding

This study was funded by the National Natural Science Foundation of China (Number: 82060462), the Science and Technology Plan Project of Guizhou Province (Number: [2019]5405), the Doctoral Foundation of Guizhou Provincial People’s Hospital (GZSYBS [2018]02), and the Science and Technology Support Plan of Guizhou Province in 2020 (No. [2020]4Y142). The funding agencies and donors had no role in any aspect of this study.

## Conflict of interest

The authors declare that the research was conducted in the absence of any commercial or financial relationships that could be construed as a potential conflict of interest.

## Publisher’s note

All claims expressed in this article are solely those of the authors and do not necessarily represent those of their affiliated organizations, or those of the publisher, the editors and the reviewers. Any product that may be evaluated in this article, or claim that may be made by its manufacturer, is not guaranteed or endorsed by the publisher.

## References

[1] Sieger N Kyriazis I Schaudinn A Kallidonis P Neuhaus J Liatsikos EN. Acute focal bacterial nephritis is associated with invasive diagnostic procedures – a cohort of 138 cases extracted through a systematic review. BMC Infect Dis (2017) 17(1):240. doi: 10.1186/s12879-017-2336-6 28376724 PMC5379728

[2] Kim MH Jun KW Hwang JK Moon IS Kim JI. Characteristics of femoral motor neuropathies induced after kidney transplantation: A case series. Transplant Proc (2016) 48(3):933–7. doi: 10.1016/j.transproceed.2015.12.072 27234771

[3] Coelho RF Schneider–Monteiro ED Mesquita JL Mazzucchi E Marmo Lucon A Srougi M. Renal and perinephric abscesses: analysis of 65 consecutive cases. World J Surg (2007) 31(2):431–6. doi: 10.1007/s00268-006-0162-x 17219288

[4] Nakamura T Morimoto T Katsube K Yamamori Y Mashino J Kikuchi K. Clinical characteristics of pyogenic spondylitis and psoas abscess at a tertiary care hospital: a retrospective cohort study. J Orthop Surg Res (2018) 13(1):302. doi: 10.1186/s13018-018-1005-9 30486831 PMC6264034

[5] Jemal A Siegel R Ward E Murray T Xu J Thun MJ. Cancer statistics, 2007. CA Cancer J Clin (2007) 7(1):43–66. doi: 10.3322/canjclin.57.1.43 17237035

[6] Kuthi L Jenei A Hajdu A Németh I Varga Z Bajory Z. Prognostic factors for renal cell carcinoma subtypes diagnosed according to the 2016 WHO renal tumor classification: a study involving 928 patients. Pathol Oncol Res (2017) 23(3):689–98. doi: 10.1007/s12253-016-0179-x 28032311

[7] Winter BM Gajda M Grimm MO. Diagnosis and treatment of retroperitoneal abscesses. Urologe A (2016) 55(6):741–7. doi: 10.1007/s00120-016-0118-1 27220893

[8] Campbell SC Clark PE Chang SS Karam JA Souter L Uzzo RG. Renal mass and localized renal cancer: Evaluation, management, and follow–up: AUA guideline: Part I. J Urol (2021) 206(2):199–208. doi: 10.1097/JU.0000000000001911 34115547

[9] Eltahawy E Kamel M Ezzet M. Management of renal cell carcinoma presenting as inflammatory renal mass. Urol Ann (2015) 7(3):330–3. doi: 10.4103/0974-7796.152051 PMC4518369 26229320

[10] Goyal A Sharma R Bhalla AS Gamanagatti S Seth A. Diffusion–weighted MRI in inflammatory renal lesions: all that glitters is not RCC! Eur Radiol (2013) 23(1):272–9. doi: 10.1007/s00330-012-2577-0 22797980

[11] Goyal A Sharma R Bhalla AS Gamanagatti S Seth A. Comparison of MDCT, MRI and MRI with diffusion–weighted imaging in evaluation of focal renal lesions: The defender, challenger, and winner! Indian J Radiol Imaging (2018) 28(1):27–36. doi: 10.4103/ijri 29692523 PMC5894314

[12] Nataluk EA McCullough DL Scharling EO. Xanthogranulomatous pyelonephritis, the gatekeeper's dilemma: a contemporary look at an old problem. Urology (1995) 45(3):377–80. doi: 10.1016/S0090-4295(99)80004-1 7879331

[13] Brown ED Brown JJ Kettritz U Shoenut JP Semelka RC. Renal abscesses: appearance on gadolinium–enhanced magnetic resonance images. Abdom Imaging (1996) 21(2):172–6. doi: 10.1007/s002619900038 8661768

[14] Kim N Juarez R Levy AD. Imaging non–vascular complications of renal transplantation. Abdom Radiol (NY) (2018) 43(10):2555–63. doi: 10.1007/s00261-018-1566-4 29550956

[15] Vici P Mariani L Pizzuti L Sergi D Di Lauro L Vizza E. Emerging biological treatments for uterine cervical carcinoma. J Cancer (2014) 5(2):86–97. doi: 10.7150/jca.7963 24494026 PMC3909763

[16] Gandhi D Hoeffner EG Carlos RC Case I Mukherji SK. Computed tomography perfusion of squamous cell carcinoma of the upper aerodigestive tract. Initial results. J Comput Assist Tomogr (2003) 27(5):687–93. doi: 10.1097/00004728-200309000-00005 14501359

[17] Li X Liang P Guo M Yu J Yu X Cheng Z. Real–time contrast–enhanced ultrasound in diagnosis of solid renal lesions. Discovery Med (2013) 16(86):15–25. doi: 10.1038/emm.2013.68 23911228

[18] Marschner CA Ruebenthaler J Schwarze V Negrão de Figueiredo G Zhang L Clevert DA. Comparison of computed tomography (CT), magnetic resonance imaging (MRI) and contrast–enhanced ultrasound (CEUS) in the evaluation of unclear renal lesions. Rofo (2020) 192(11):1053–9. doi: 10.1055/a-1127-3371 32294790

[19] Roarke MC Collins JM Nguyen BD. Indolent enterococcal abscess mimicking recurrent renal cell carcinoma on MR imaging and PET/CT after radiofrequency ablation. J Vasc Interv Radiol (2006) 17(11 Pt 1):1851–4. doi: 10.1097/01.RVI.0000242169.99178.CB 17142718

[20] Blake MA McKernan M Setty B Fischman AJ Mueller PR. Renal oncocytoma displaying intense activity on 18F–FDG PET. AJR Am J Roentgenol (2006) 186(1):269–70. doi: 10.2214/AJR.05.0110 16357422

[21] Marshall FF. Efficiency of [18F] FDG PET in characterising renal cancer and detecting distant metastases: a comparison with CT. J Urol (2005) 173(3):730. doi: 10.1016/s0022-5347(05)60320-5 15711256

[22] Leveridge MJ Finelli A Kachura JR Evans A Chung H Shiff DA. Outcomes of small renal mass needle core biopsy, nondiagnostic percutaneous biopsy, and the role of repeat biopsy. Eur Urol (2011) 60(3):578–84. doi: 10.1016/j.eururo.2011.06.021 21704449

[23] Pandharipande PV Gervais DA Hartman RI Harisinghani MG Feldman AS Mueller PR. Renal mass biopsy to guide treatment decisions for small incidental renal tumors: a cost–effectiveness analysis. Radiology (2010) 256(3):836–46. doi: 10.1148/radiol.10092013 PMC2923731 20720070

[24] Perrino CM Cramer HM Chen S Idrees MT Wu HH. World health organization (WHO)/International society of urological pathology (ISUP) grading in fine–needle aspiration biopsies of renal masses. Diagn Cytopathol (2018) 46(11):895–900. doi: 10.1002/dc.23979 30488673

[25] Walsh P Retik A Vaughan E Wein A. Infections of the urinary tract. In: Aufl. campbell’s urology, 8. Amsterdam: Elsevier (2002). p. S515–592.

[26] Conan PL Podglajen I Compain F Osman M Lebeaux D Flamarion E. Renal abscess caused by panton–valentine leukocidin–producing staphylococcus aureus: report of an unusual case and review of the literature. Infect Dis (Lond) (2021) 53(2):131–6. doi: 10.1080/23744235.2020.1856920 33307902

[27] Udare A Abreu–Gomez J Krishna S McInnes M Siegelman E Schieda N. Imaging manifestations of acute and chronic renal infection that mimics malignancy: How to make the diagnosis using computed tomography and magnetic resonance imaging. Can Assoc Radiol J (2019) 70(4):424–33. doi: 10.1016/j.carj.2019.07.002 31537315

[28] Kondo T Nakazawa H Sakai F Kuwata T Onitsuka S Hashimoto Y. Spoke–wheel–like enhancement as an important imaging finding of chromophobe cell renal carcinoma: a retrospective analysis on computed tomography and magnetic resonance imaging studies. Int J Urol (2004) 11(10):817–24. doi: 10.1111/j.1442-2042.2004.0090 15479284

